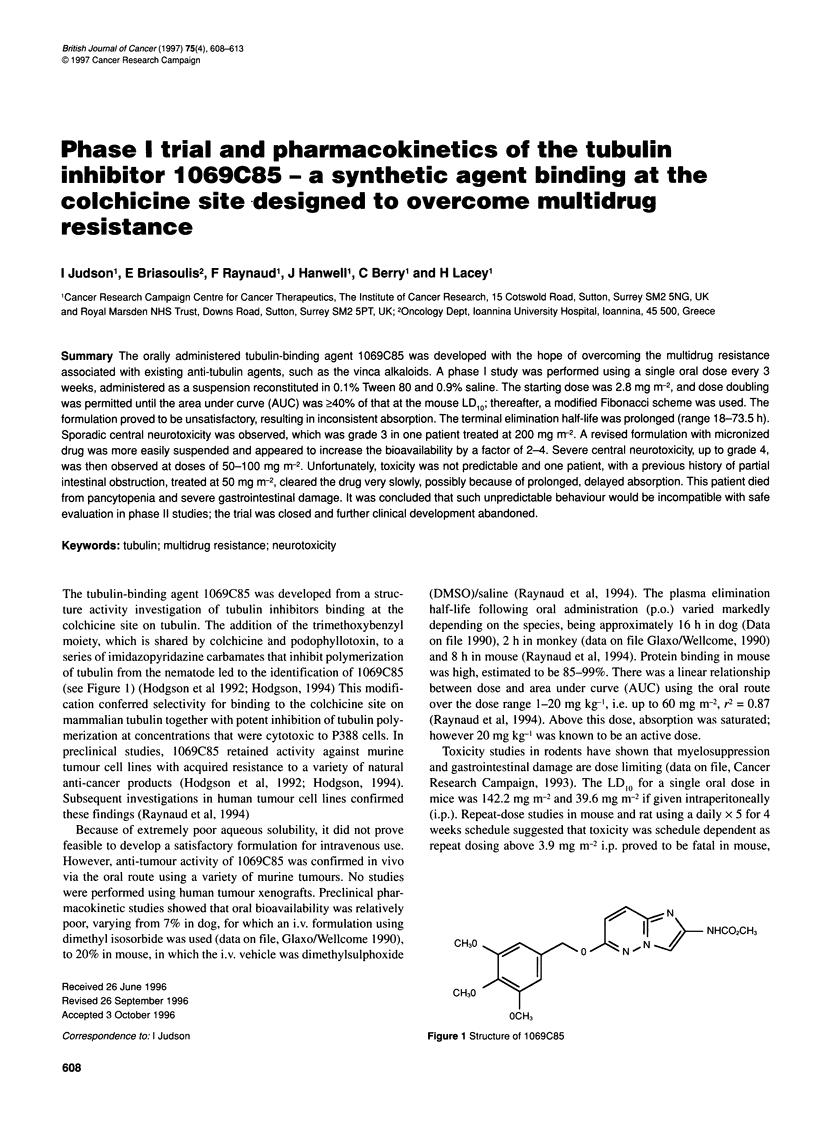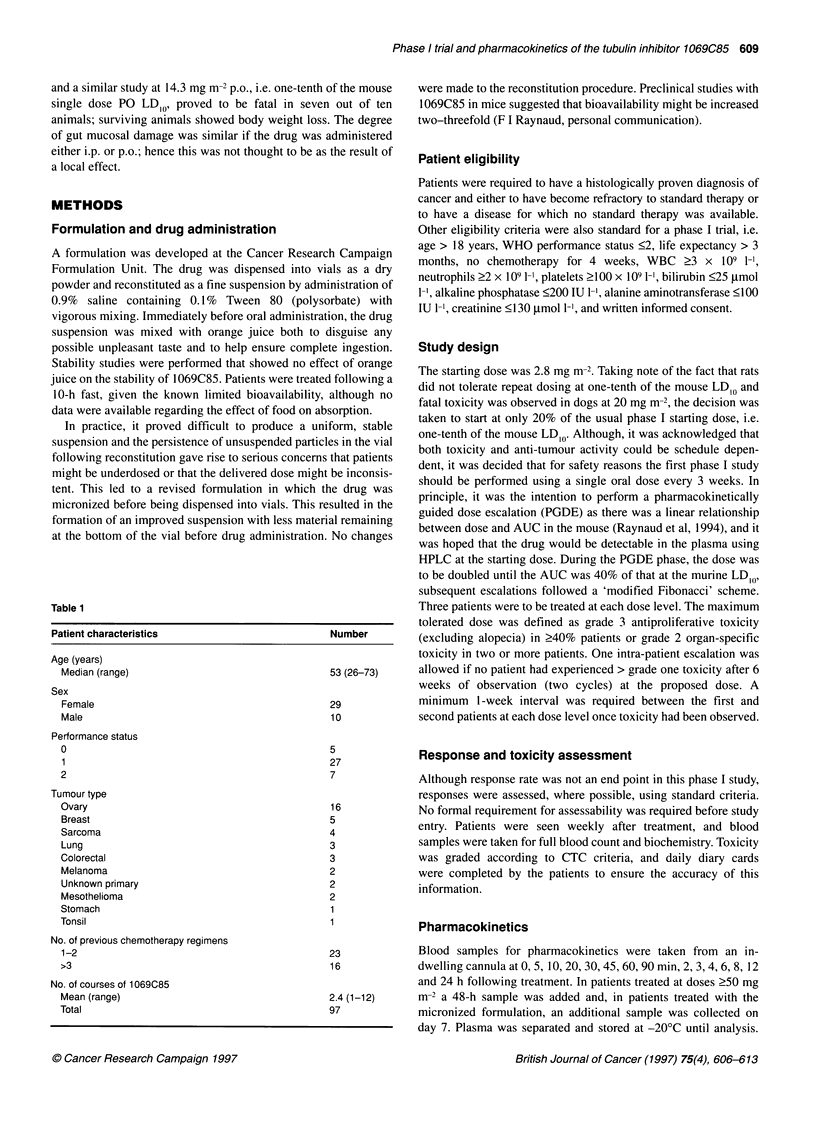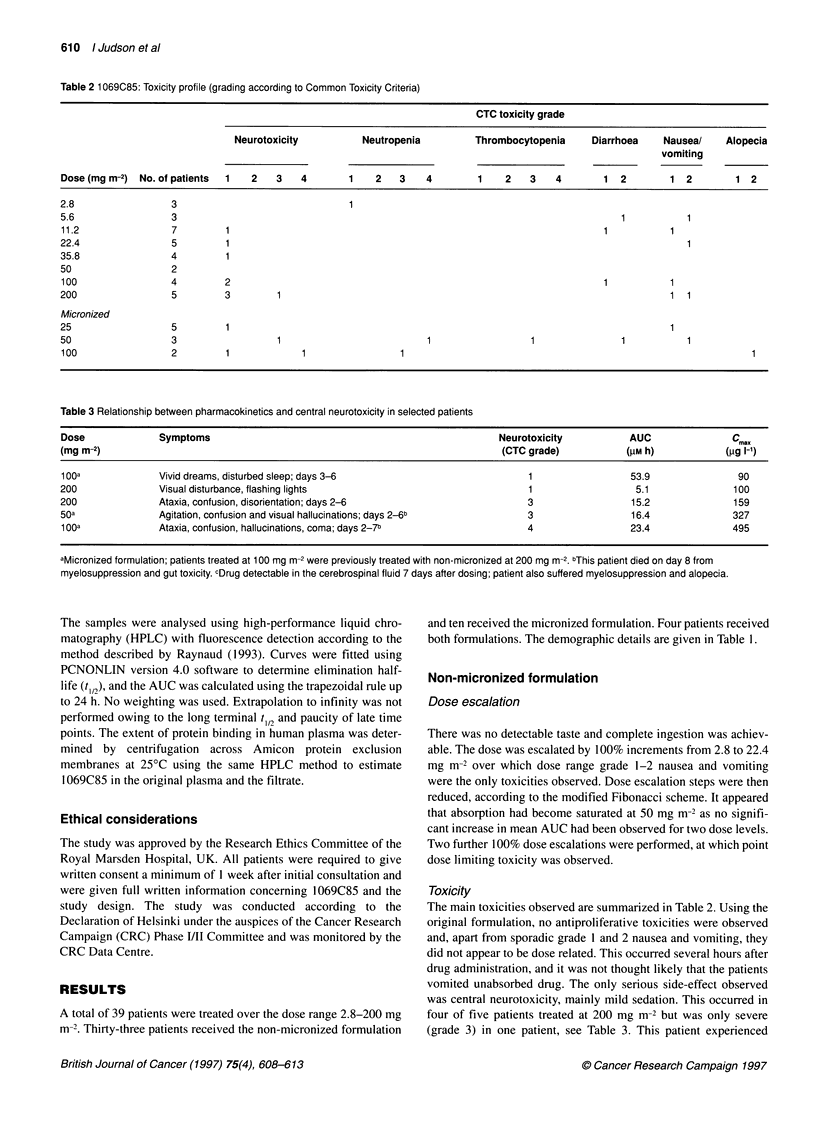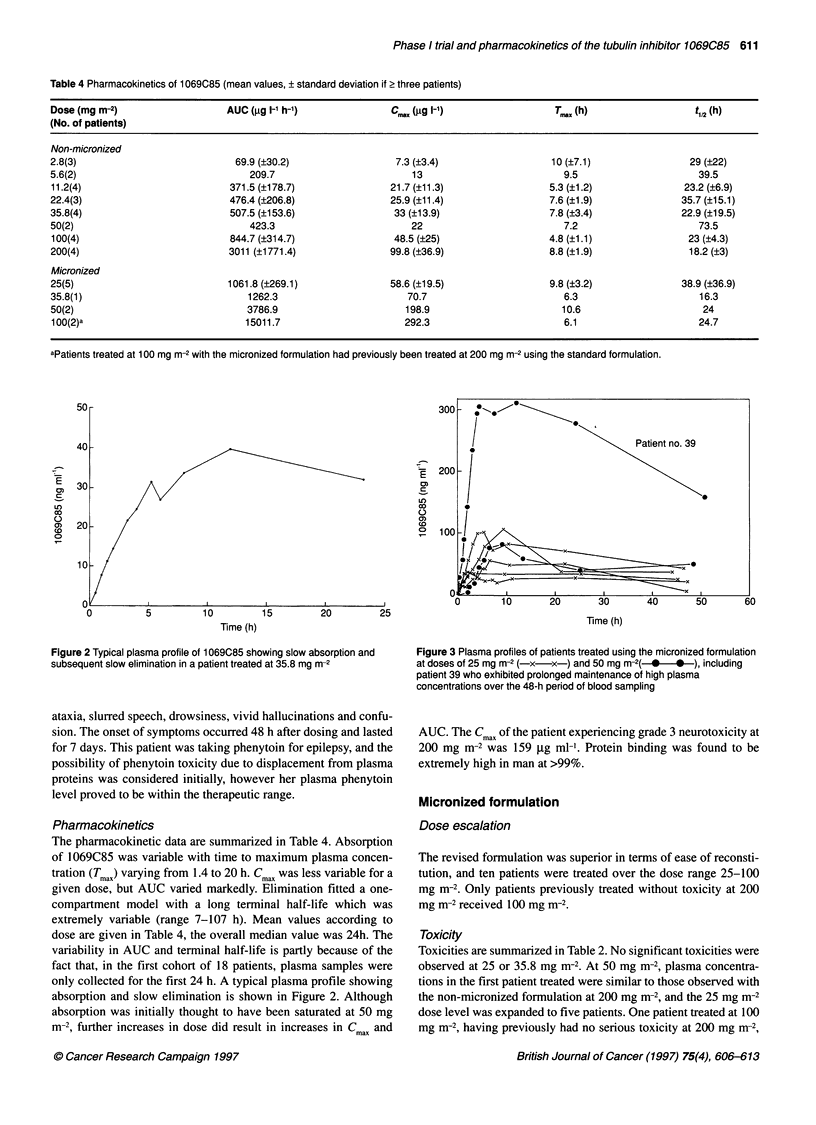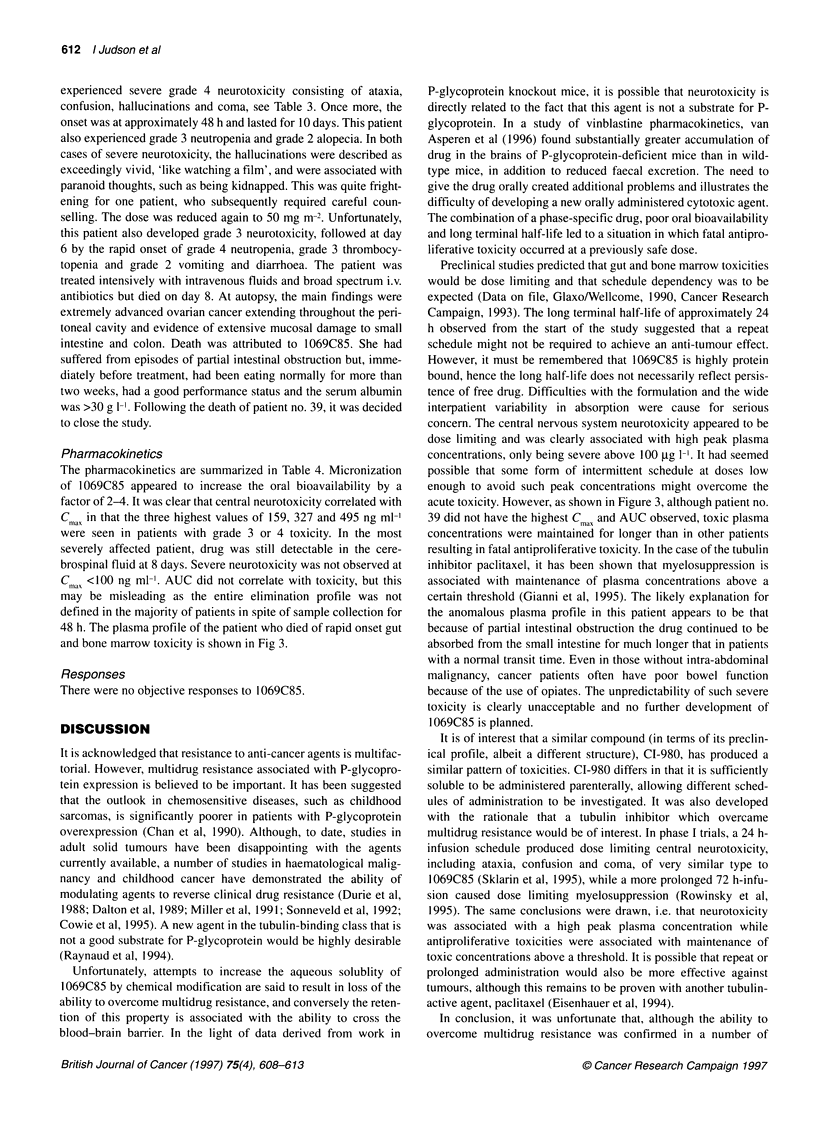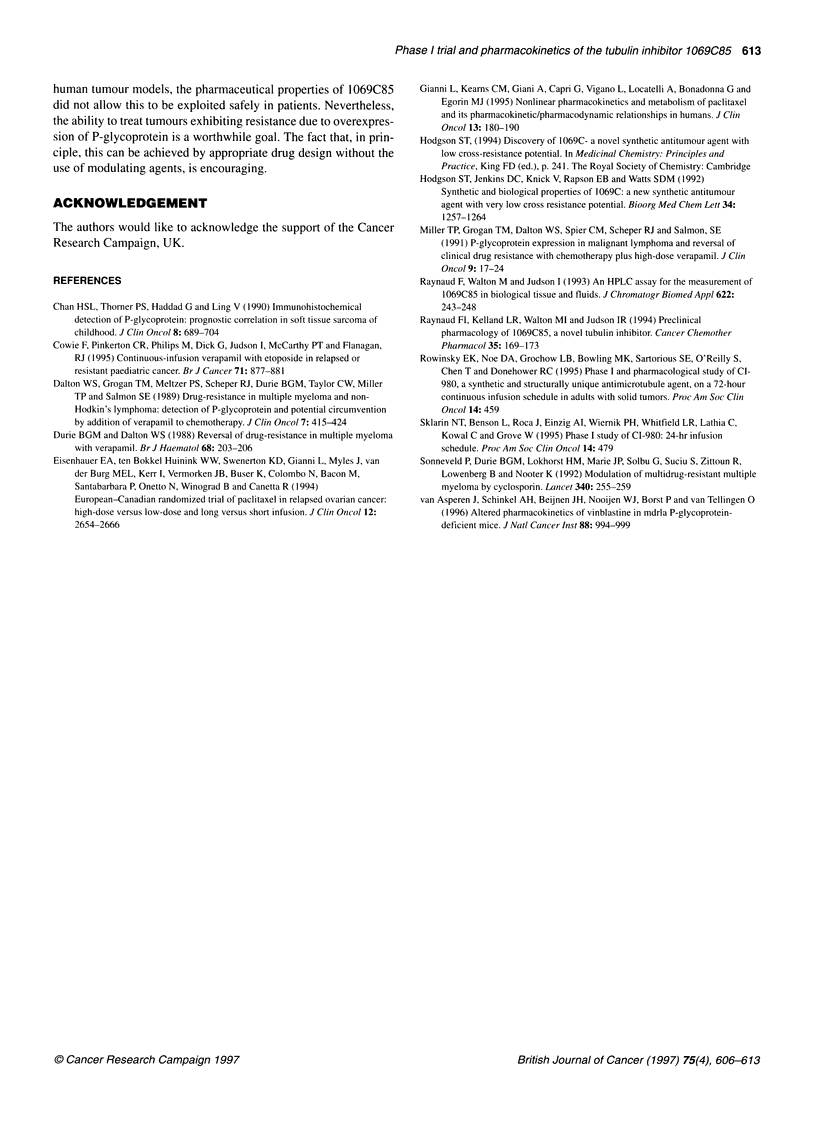# Phase I trial and pharmacokinetics of the tubulin inhibitor 1069C85--a synthetic agent binding at the colchicine site designed to overcome multidrug resistance.

**DOI:** 10.1038/bjc.1997.107

**Published:** 1997

**Authors:** I. Judson, E. Briasoulis, F. Raynaud, J. Hanwell, C. Berry, H. Lacey

**Affiliations:** Cancer Research Campaign Centre for Cancer Therapeutics, Institute of Cancer Research, Sutton, Surrey, UK.

## Abstract

The orally administered tubulin-binding agent 1069C85 was developed with the hope of overcoming the multidrug resistance associated with existing anti-tubulin agents, such as the vinca alkaloids. A phase I study was performed using a single oral dose every 3 weeks, administered as a suspension reconstituted in 0.1% Tween 80 and 0.9% saline. The starting dose was 2.8 mg m-2, and dose doubling was permitted until the area under curve (AUC) was > or = 40% of that at the mouse LD10; thereafter, a modified Fibonacci scheme was used. The formulation proved to be unsatisfactory, resulting in inconsistent absorption. The terminal elimination half-life was prolonged (range 18-73.5 h). Sporadic central neurotoxicity was observed, which was grade 3 in one patient treated at 200 mg m-2. A revised formulation with micronized drug was more easily suspended and appeared to increase the bioavailability by a factor of 2-4. Severe central neurotoxicity, up to grade 4, was then observed at doses of 50-100 mg m-2. Unfortunately, toxicity was not predictable and one patient, with a previous history of partial intestinal obstruction, treated at 50 mg m-2, cleared the drug very slowly, possibly because of prolonged, delayed absorption. This patient died from pancytopenia and severe gastrointestinal damage. It was concluded that such unpredictable behaviour would be incompatible with safe evaluation in phase II studies; the trial was closed and further clinical development abandoned.


British Journal of Cancer (1997) 75(4), 608–613
© 1997 Cancer Research Campaign

### Phase I trial and pharmacokinetics of the tubulin inhibitor 1069C85 – a synthetic agent binding at the colchicine site designed to overcome multidrug resistance

**I Judson[1], E Briasoulis[2], F Raynaud[1], J Hanwell[1], C Berry[1] and H Lacey[1]**

[1]Cancer Research Campaign Centre for Cancer Therapeutics, The Institute of Cancer Research, 15 Cotswold Road, Sutton, Surrey SM2 5NG, UK and Royal Marsden NHS Trust, Downs Road, Sutton, Surrey SM2 5PT, UK; [2]Oncology Dept, Ioannina University Hospital, Ioannina, 45 500, Greece

**Summary** The orally administered tubulin-binding agent 1069C85 was developed with the hope of overcoming the multidrug resistance associated with existing anti-tubulin agents, such as the vinca alkaloids. A phase I study was performed using a single oral dose every 3 weeks, administered as a suspension reconstituted in 0.1% Tween 80 and 0.9% saline. The starting dose was 2.8 mg $m^{-2}$, and dose doubling was permitted until the area under curve (AUC) was ≥40% of that at the mouse $LD_{10}$; thereafter, a modified Fibonacci scheme was used. The formulation proved to be unsatisfactory, resulting in inconsistent absorption. The terminal elimination half-life was prolonged (range 18–73.5 h). Sporadic central neurotoxicity was observed, which was grade 3 in one patient treated at 200 mg $m^{-2}$. A revised formulation with micronized drug was more easily suspended and appeared to increase the bioavailability by a factor of 2–4. Severe central neurotoxicity, up to grade 4, was then observed at doses of 50–100 mg $m^{-2}$. Unfortunately, toxicity was not predictable and one patient, with a previous history of partial intestinal obstruction, treated at 50 mg $m^{-2}$, cleared the drug very slowly, possibly because of prolonged, delayed absorption. This patient died from pancytopenia and severe gastrointestinal damage. It was concluded that such unpredictable behaviour would be incompatible with safe evaluation in phase II studies; the trial was closed and further clinical development abandoned.

**Keywords:** tubulin; multidrug resistance; neurotoxicity

The tubulin-binding agent 1069C85 was developed from a structure activity investigation of tubulin inhibitors binding at the colchicine site on tubulin. The addition of the trimethoxybenzyl moiety, which is shared by colchicine and podophyllotoxin, to a series of imidazopyridazine carbamates that inhibit polymerization of tubulin from the nematode led to the identification of 1069C85 (see Figure 1) (Hodgson et al 1992; Hodgson, 1994) This modification conferred selectivity for binding to the colchicine site on mammalian tubulin together with potent inhibition of tubulin polymerization at concentrations that were cytotoxic to P388 cells. In preclinical studies, 1069C85 retained activity against murine tumour cell lines with acquired resistance to a variety of natural anti-cancer products (Hodgson et al, 1992; Hodgson, 1994). Subsequent investigations in human tumour cell lines confirmed these findings (Raynaud et al, 1994)

Because of extremely poor aqueous solubility, it did not prove feasible to develop a satisfactory formulation for intravenous use. However, anti-tumour activity of 1069C85 was confirmed in vivo via the oral route using a variety of murine tumours. No studies were performed using human tumour xenografts. Preclinical pharmacokinetic studies showed that oral bioavailability was relatively poor, varying from 7% in dog, for which an i.v. formulation using dimethyl isosorbide was used (data on file, Glaxo/Wellcome 1990), to 20% in mouse, in which the i.v. vehicle was dimethylsulphoxide (DMSO)/saline (Raynaud et al, 1994). The plasma elimination half-life following oral administration (p.o.) varied markedly depending on the species, being approximately 16 h in dog (Data on file 1990), 2 h in monkey (data on file Glaxo/Wellcome, 1990) and 8 h in mouse (Raynaud et al, 1994). Protein binding in mouse was high, estimated to be 85–99%. There was a linear relationship between dose and area under curve (AUC) using the oral route over the dose range 1–20 mg $kg^{-1}$, i.e. up to 60 mg $m^{-2}$, $r^2 = 0.87$ (Raynaud et al, 1994). Above this dose, absorption was saturated; however 20 mg $kg^{-1}$ was known to be an active dose.

Toxicity studies in rodents have shown that myelosuppression and gastrointestinal damage are dose limiting (data on file, Cancer Research Campaign, 1993). The $LD_{10}$ for a single oral dose in mice was 142.2 mg $m^{-2}$ and 39.6 mg $m^{-2}$ if given intraperitoneally (i.p.). Repeat-dose studies in mouse and rat using a daily × 5 for 4 weeks schedule suggested that toxicity was schedule dependent as repeat dosing above 3.9 mg $m^{-2}$ i.p. proved to be fatal in mouse,

**Figure 1** Structure of 1069C85

Received 26 June 1996
Revised 26 September 1996
Accepted 3 October 1996

*Correspondence to:* I Judson and a similar study at 14.3 mg m$^{-2}$ p.o., i.e. one-tenth of the mouse single dose PO $LD_{10}$, proved to be fatal in seven out of ten animals; surviving animals showed body weight loss. The degree of gut mucosal damage was similar if the drug was administered either i.p. or p.o.; hence this was not thought to be as the result of a local effect.

### METHODS

### Formulation and drug administration

A formulation was developed at the Cancer Research Campaign Formulation Unit. The drug was dispensed into vials as a dry powder and reconstituted as a fine suspension by administration of 0.9% saline containing 0.1% Tween 80 (polysorbate) with vigorous mixing. Immediately before oral administration, the drug suspension was mixed with orange juice both to disguise any possible unpleasant taste and to help ensure complete ingestion. Stability studies were performed that showed no effect of orange juice on the stability of 1069C85. Patients were treated following a 10-h fast, given the known limited bioavailability, although no data were available regarding the effect of food on absorption.

In practice, it proved difficult to produce a uniform, stable suspension and the persistence of unsuspended particles in the vial following reconstitution gave rise to serious concerns that patients might be underdosed or that the delivered dose might be inconsistent. This led to a revised formulation in which the drug was micronized before being dispensed into vials. This resulted in the formation of an improved suspension with less material remaining at the bottom of the vial before drug administration. No changes were made to the reconstitution procedure. Preclinical studies with 1069C85 in mice suggested that bioavailability might be increased two–threefold (F I Raynaud, personal communication).

**Table 1**

| Patient characteristics | Number |
|---|---|
| Age (years) | |
| Median (range) | 53 (26–73) |
| Sex | |
| Female | 29 |
| Male | 10 |
| Performance status | |
| 0 | 5 |
| 1 | 27 |
| 2 | 7 |
| Tumour type | |
| Ovary | 16 |
| Breast | 5 |
| Sarcoma | 4 |
| Lung | 3 |
| Colorectal | 3 |
| Melanoma | 2 |
| Unknown primary | 2 |
| Mesothelioma | 2 |
| Stomach | 1 |
| Tonsil | 1 |
| No. of previous chemotherapy regimens | |
| 1–2 | 23 |
| >3 | 16 |
| No. of courses of 1069C85 | |
| Mean (range) | 2.4 (1–12) |
| Total | 97 |

### Patient eligibility

Patients were required to have a histologically proven diagnosis of cancer and either to have become refractory to standard therapy or to have a disease for which no standard therapy was available. Other eligibility criteria were also standard for a phase I trial, i.e. age > 18 years, WHO performance status ≤2, life expectancy > 3 months, no chemotherapy for 4 weeks, WBC ≥3 × 10$^{9}$ l$^{-1}$, neutrophils ≥2 × 10$^{9}$ l$^{-1}$, platelets ≥100 × 10$^{9}$ l$^{-1}$, bilirubin ≤25 μmol l$^{-1}$, alkaline phosphatase ≤200 IU l$^{-1}$, alanine aminotransferase ≤100 IU l$^{-1}$, creatinine ≤130 μmol l$^{-1}$, and written informed consent.

### Study design

The starting dose was 2.8 mg m$^{-2}$. Taking note of the fact that rats did not tolerate repeat dosing at one-tenth of the mouse $LD_{10}$ and fatal toxicity was observed in dogs at 20 mg m$^{-2}$, the decision was taken to start at only 20% of the usual phase I starting dose, i.e. one-tenth of the mouse $LD_{10}$. Although, it was acknowledged that both toxicity and anti-tumour activity could be schedule dependent, it was decided that for safety reasons the first phase I study should be performed using a single oral dose every 3 weeks. In principle, it was the intention to perform a pharmacokinetically guided dose escalation (PGDE) as there was a linear relationship between dose and AUC in the mouse (Raynaud et al, 1994), and it was hoped that the drug would be detectable in the plasma using HPLC at the starting dose. During the PGDE phase, the dose was to be doubled until the AUC was 40% of that at the murine $LD_{10}$, subsequent escalations followed a 'modified Fibonacci' scheme. Three patients were to be treated at each dose level. The maximum tolerated dose was defined as grade 3 antiproliferative toxicity (excluding alopecia) in ≥40% patients or grade 2 organ-specific toxicity in two or more patients. One intra-patient escalation was allowed if no patient had experienced > grade one toxicity after 6 weeks of observation (two cycles) at the proposed dose. A minimum 1-week interval was required between the first and second patients at each dose level once toxicity had been observed.

### Response and toxicity assessment

Although response rate was not an end point in this phase I study, responses were assessed, where possible, using standard criteria. No formal requirement for assessability was required before study entry. Patients were seen weekly after treatment, and blood samples were taken for full blood count and biochemistry. Toxicity was graded according to CTC criteria, and daily diary cards were completed by the patients to ensure the accuracy of this information.

### Pharmacokinetics

Blood samples for pharmacokinetics were taken from an indwelling cannula at 0, 5, 10, 20, 30, 45, 60, 90 min, 2, 3, 4, 6, 8, 12 and 24 h following treatment. In patients treated at doses ≥50 mg m$^{-2}$ a 48-h sample was added and, in patients treated with the micronized formulation, an additional sample was collected on day 7. Plasma was separated and stored at –20°C until analysis.

© Cancer Research Campaign 1997

**Table 2** 1069C85: Toxicity profile (grading according to Common Toxicity Criteria)

| | | CTC toxicity grade | | | | | | | | | | | | | | | | | |
|---|---|---|---|---|---|---|---|---|---|---|---|---|---|---|---|---|---|---|---|
| | | Neurotoxicity | | | | Neutropenia | | | | Thrombocytopenia | | | | Diarrhoea | | Nausea/ vomiting | | Alopecia | |
| Dose (mg $m^{-2}$) | No. of patients | 1 | 2 | 3 | 4 | 1 | 2 | 3 | 4 | 1 | 2 | 3 | 4 | 1 | 2 | 1 | 2 | 1 | 2 |
| 2.8 | 3 | | | | | 1 | | | | | | | | | | | | | |
| 5.6 | 3 | | | | | | | | | | | | | | 1 | | 1 | | |
| 11.2 | 7 | 1 | | | | | | | | | | | | 1 | | 1 | | | |
| 22.4 | 5 | 1 | | | | | | | | | | | | | | | 1 | | |
| 35.8 | 4 | 1 | | | | | | | | | | | | | | | | | |
| 50 | 2 | | | | | | | | | | | | | | | | | | |
| 100 | 4 | 2 | | | | | | | | | | | | 1 | | 1 | | | |
| 200 | 5 | 3 | | 1 | | | | | | | | | | | | 1 | 1 | | |
| *Micronized* | | | | | | | | | | | | | | | | | | | |
| 25 | 5 | 1 | | | | | | | | | | | | | | 1 | | | |
| 50 | 3 | | | 1 | | | | | 1 | | | 1 | | | 1 | | 1 | | |
| 100 | 2 | 1 | | | 1 | | | 1 | | | | | | | | | | | 1 |

**Table 3** Relationship between pharmacokinetics and central neurotoxicity in selected patients

| Dose (mg $m^{-2}$) | Symptoms | Neurotoxicity (CTC grade) | AUC (μM h) | $C_{max}$ (μg $l^{-1}$) |
|---|---|---|---|---|
| 100[a] | Vivid dreams, disturbed sleep; days 3–6 | 1 | 53.9 | 90 |
| 200 | Visual disturbance, flashing lights | 1 | 5.1 | 100 |
| 200 | Ataxia, confusion, disorientation; days 2–6 | 3 | 15.2 | 159 |
| 50[a] | Agitation, confusion and visual hallucinations; days 2–6[b] | 3 | 16.4 | 327 |
| 100[a] | Ataxia, confusion, hallucinations, coma; days 2–7[b] | 4 | 23.4 | 495 |

[a]Micronized formulation; patients treated at 100 mg $m^{-2}$ were previously treated with non-micronized at 200 mg $m^{-2}$. [b]This patient died on day 8 from myelosuppression and gut toxicity. [c]Drug detectable in the cerebrospinal fluid 7 days after dosing; patient also suffered myelosuppression and alopecia.

The samples were analysed using high-performance liquid chromatography (HPLC) with fluorescence detection according to the method described by Raynaud (1993). Curves were fitted using PCNONLIN version 4.0 software to determine elimination half-life ($t_{1/2}$), and the AUC was calculated using the trapezoidal rule up to 24 h. No weighting was used. Extrapolation to infinity was not performed owing to the long terminal $t_{1/2}$ and paucity of late time points. The extent of protein binding in human plasma was determined by centrifugation across Amicon protein exclusion membranes at 25°C using the same HPLC method to estimate 1069C85 in the original plasma and the filtrate.

### Ethical considerations

The study was approved by the Research Ethics Committee of the Royal Marsden Hospital, UK. All patients were required to give written consent a minimum of 1 week after initial consultation and were given full written information concerning 1069C85 and the study design. The study was conducted according to the Declaration of Helsinki under the auspices of the Cancer Research Campaign (CRC) Phase I/II Committee and was monitored by the CRC Data Centre.

### RESULTS

A total of 39 patients were treated over the dose range 2.8–200 mg $m^{-2}$. Thirty-three patients received the non-micronized formulation and ten received the micronized formulation. Four patients received both formulations. The demographic details are given in Table 1.

### Non-micronized formulation

### *Dose escalation*

There was no detectable taste and complete ingestion was achievable. The dose was escalated by 100% increments from 2.8 to 22.4 mg $m^{-2}$ over which dose range grade 1–2 nausea and vomiting were the only toxicities observed. Dose escalation steps were then reduced, according to the modified Fibonacci scheme. It appeared that absorption had become saturated at 50 mg $m^{-2}$ as no significant increase in mean AUC had been observed for two dose levels. Two further 100% dose escalations were performed, at which point dose limiting toxicity was observed.

### *Toxicity*

The main toxicities observed are summarized in Table 2. Using the original formulation, no antiproliferative toxicities were observed and, apart from sporadic grade 1 and 2 nausea and vomiting, they did not appear to be dose related. This occurred several hours after drug administration, and it was not thought likely that the patients vomited unabsorbed drug. The only serious side-effect observed was central neurotoxicity, mainly mild sedation. This occurred in four of five patients treated at 200 mg $m^{-2}$ but was only severe (grade 3) in one patient, see Table 3. This patient experienced

© Cancer Research Campaign 1997

**Table 4** Pharmacokinetics of 1069C85 (mean values, ± standard deviation if ≥ three patients)

| Dose (mg m$^{-2}$) (No. of patients) | AUC (μg l$^{-1}$ h$^{-1}$) | $C_{max}$ (μg l$^{-1}$) | $T_{max}$ (h) | $t_{1/2}$ (h) |
|---|---|---|---|---|
| *Non-micronized* | | | | |
| 2.8(3) | 69.9 (±30.2) | 7.3 (±3.4) | 10 (±7.1) | 29 (±22) |
| 5.6(2) | 209.7 | 13 | 9.5 | 39.5 |
| 11.2(4) | 371.5 (±178.7) | 21.7 (±11.3) | 5.3 (±1.2) | 23.2 (±6.9) |
| 22.4(3) | 476.4 (±206.8) | 25.9 (±11.4) | 7.6 (±1.9) | 35.7 (±15.1) |
| 35.8(4) | 507.5 (±153.6) | 33 (±13.9) | 7.8 (±3.4) | 22.9 (±19.5) |
| 50(2) | 423.3 | 22 | 7.2 | 73.5 |
| 100(4) | 844.7 (±314.7) | 48.5 (±25) | 4.8 (±1.1) | 23 (±4.3) |
| 200(4) | 3011 (±1771.4) | 99.8 (±36.9) | 8.8 (±1.9) | 18.2 (±3) |
| *Micronized* | | | | |
| 25(5) | 1061.8 (±269.1) | 58.6 (±19.5) | 9.8 (±3.2) | 38.9 (±36.9) |
| 35.8(1) | 1262.3 | 70.7 | 6.3 | 16.3 |
| 50(2) | 3786.9 | 198.9 | 10.6 | 24 |
| 100(2)[a] | 15011.7 | 292.3 | 6.1 | 24.7 |

[a]Patients treated at 100 mg m$^{-2}$ with the micronized formulation had previously been treated at 200 mg m$^{-2}$ using the standard formulation.

**Figure 2** Typical plasma profile of 1069C85 showing slow absorption and subsequent slow elimination in a patient treated at 35.8 mg m$^{-2}$

**Figure 3** Plasma profiles of patients treated using the micronized formulation at doses of 25 mg m$^{-2}$ (—x——x—) and 50 mg m$^{-2}$(—●——●—), including patient 39 who exhibited prolonged maintenance of high plasma concentrations over the 48-h period of blood sampling ataxia, slurred speech, drowsiness, vivid hallucinations and confusion. The onset of symptoms occurred 48 h after dosing and lasted for 7 days. This patient was taking phenytoin for epilepsy, and the possibility of phenytoin toxicity due to displacement from plasma proteins was considered initially, however her plasma phenytoin level proved to be within the therapeutic range.

### *Pharmacokinetics*

The pharmacokinetic data are summarized in Table 4. Absorption of 1069C85 was variable with time to maximum plasma concentration ($T_{max}$) varying from 1.4 to 20 h. $C_{max}$ was less variable for a given dose, but AUC varied markedly. Elimination fitted a one-compartment model with a long terminal half-life which was extremely variable (range 7–107 h). Mean values according to dose are given in Table 4, the overall median value was 24h. The variability in AUC and terminal half-life is partly because of the fact that, in the first cohort of 18 patients, plasma samples were only collected for the first 24 h. A typical plasma profile showing absorption and slow elimination is shown in Figure 2. Although absorption was initially thought to have been saturated at 50 mg m$^{-2}$, further increases in dose did result in increases in $C_{max}$ and AUC. The $C_{max}$ of the patient experiencing grade 3 neurotoxicity at 200 mg m$^{-2}$ was 159 μg ml$^{-1}$. Protein binding was found to be extremely high in man at >99%.

### Micronized formulation

### *Dose escalation*

The revised formulation was superior in terms of ease of reconstitution, and ten patients were treated over the dose range 25–100 mg m$^{-2}$. Only patients previously treated without toxicity at 200 mg m$^{-2}$ received 100 mg m$^{-2}$.

### *Toxicity*

Toxicities are summarized in Table 2. No significant toxicities were observed at 25 or 35.8 mg m$^{-2}$. At 50 mg m$^{-2}$, plasma concentrations in the first patient treated were similar to those observed with the non-micronized formulation at 200 mg m$^{-2}$, and the 25 mg m$^{-2}$ dose level was expanded to five patients. One patient treated at 100 mg m$^{-2}$, having previously had no serious toxicity at 200 mg m$^{-2}$, experienced severe grade 4 neurotoxicity consisting of ataxia, confusion, hallucinations and coma, see Table 3. Once more, the onset was at approximately 48 h and lasted for 10 days. This patient also experienced grade 3 neutropenia and grade 2 alopecia. In both cases of severe neurotoxicity, the hallucinations were described as exceedingly vivid, 'like watching a film', and were associated with paranoid thoughts, such as being kidnapped. This was quite frightening for one patient, who subsequently required careful counselling. The dose was reduced again to 50 mg $m^{-2}$. Unfortunately, this patient also developed grade 3 neurotoxicity, followed at day 6 by the rapid onset of grade 4 neutropenia, grade 3 thrombocytopenia and grade 2 vomiting and diarrhoea. The patient was treated intensively with intravenous fluids and broad spectrum i.v. antibiotics but died on day 8. At autopsy, the main findings were extremely advanced ovarian cancer extending throughout the peritoneal cavity and evidence of extensive mucosal damage to small intestine and colon. Death was attributed to 1069C85. She had suffered from episodes of partial intestinal obstruction but, immediately before treatment, had been eating normally for more than two weeks, had a good performance status and the serum albumin was >30 g $l^{-1}$. Following the death of patient no. 39, it was decided to close the study.

### Pharmacokinetics

The pharmacokinetics are summarized in Table 4. Micronization of 1069C85 appeared to increase the oral bioavailability by a factor of 2–4. It was clear that central neurotoxicity correlated with $C_{max}$ in that the three highest values of 159, 327 and 495 ng $ml^{-1}$ were seen in patients with grade 3 or 4 toxicity. In the most severely affected patient, drug was still detectable in the cerebrospinal fluid at 8 days. Severe neurotoxicity was not observed at $C_{max}$ <100 ng $ml^{-1}$. AUC did not correlate with toxicity, but this may be misleading as the entire elimination profile was not defined in the majority of patients in spite of sample collection for 48 h. The plasma profile of the patient who died of rapid onset gut and bone marrow toxicity is shown in Fig 3.

### Responses

There were no objective responses to 1069C85.

### DISCUSSION

It is acknowledged that resistance to anti-cancer agents is multifactorial. However, multidrug resistance associated with P-glycoprotein expression is believed to be important. It has been suggested that the outlook in chemosensitive diseases, such as childhood sarcomas, is significantly poorer in patients with P-glycoprotein overexpression (Chan et al, 1990). Although, to date, studies in adult solid tumours have been disappointing with the agents currently available, a number of studies in haematological malignancy and childhood cancer have demonstrated the ability of modulating agents to reverse clinical drug resistance (Durie et al, 1988; Dalton et al, 1989; Miller et al, 1991; Sonneveld et al, 1992; Cowie et al, 1995). A new agent in the tubulin-binding class that is not a good substrate for P-glycoprotein would be highly desirable (Raynaud et al, 1994).

Unfortunately, attempts to increase the aqueous solublity of 1069C85 by chemical modification are said to result in loss of the ability to overcome multidrug resistance, and conversely the retention of this property is associated with the ability to cross the blood–brain barrier. In the light of data derived from work in P-glycoprotein knockout mice, it is possible that neurotoxicity is directly related to the fact that this agent is not a substrate for P-glycoprotein. In a study of vinblastine pharmacokinetics, van Asperen et al (1996) found substantially greater accumulation of drug in the brains of P-glycoprotein-deficient mice than in wild-type mice, in addition to reduced faecal excretion. The need to give the drug orally created additional problems and illustrates the difficulty of developing a new orally administered cytotoxic agent. The combination of a phase-specific drug, poor oral bioavailability and long terminal half-life led to a situation in which fatal antiproliferative toxicity occurred at a previously safe dose.

Preclinical studies predicted that gut and bone marrow toxicities would be dose limiting and that schedule dependency was to be expected (Data on file, Glaxo/Wellcome, 1990, Cancer Research Campaign, 1993). The long terminal half-life of approximately 24 h observed from the start of the study suggested that a repeat schedule might not be required to achieve an anti-tumour effect. However, it must be remembered that 1069C85 is highly protein bound, hence the long half-life does not necessarily reflect persistence of free drug. Difficulties with the formulation and the wide interpatient variability in absorption were cause for serious concern. The central nervous system neurotoxicity appeared to be dose limiting and was clearly associated with high peak plasma concentrations, only being severe above 100 μg $l^{-1}$. It had seemed possible that some form of intermittent schedule at doses low enough to avoid such peak concentrations might overcome the acute toxicity. However, as shown in Figure 3, although patient no. 39 did not have the highest $C_{max}$ and AUC observed, toxic plasma concentrations were maintained for longer than in other patients resulting in fatal antiproliferative toxicity. In the case of the tubulin inhibitor paclitaxel, it has been shown that myelosuppression is associated with maintenance of plasma concentrations above a certain threshold (Gianni et al, 1995). The likely explanation for the anomalous plasma profile in this patient appears to be that because of partial intestinal obstruction the drug continued to be absorbed from the small intestine for much longer that in patients with a normal transit time. Even in those without intra-abdominal malignancy, cancer patients often have poor bowel function because of the use of opiates. The unpredictability of such severe toxicity is clearly unacceptable and no further development of 1069C85 is planned.

It is of interest that a similar compound (in terms of its preclinical profile, albeit a different structure), CI-980, has produced a similar pattern of toxicities. CI-980 differs in that it is sufficiently soluble to be administered parenterally, allowing different schedules of administration to be investigated. It was also developed with the rationale that a tubulin inhibitor which overcame multidrug resistance would be of interest. In phase I trials, a 24 h-infusion schedule produced dose limiting central neurotoxicity, including ataxia, confusion and coma, of very similar type to 1069C85 (Sklarin et al, 1995), while a more prolonged 72 h-infusion caused dose limiting myelosuppression (Rowinsky et al, 1995). The same conclusions were drawn, i.e. that neurotoxicity was associated with a high peak plasma concentration while antiproliferative toxicities were associated with maintenance of toxic concentrations above a threshold. It is possible that repeat or prolonged administration would also be more effective against tumours, although this remains to be proven with another tubulin-active agent, paclitaxel (Eisenhauer et al, 1994).

In conclusion, it was unfortunate that, although the ability to overcome multidrug resistance was confirmed in a number of

© Cancer Research Campaign 1997

human tumour models, the pharmaceutical properties of 1069C85 did not allow this to be exploited safely in patients. Nevertheless, the ability to treat tumours exhibiting resistance due to overexpression of P-glycoprotein is a worthwhile goal. The fact that, in principle, this can be achieved by appropriate drug design without the use of modulating agents, is encouraging.

### ACKNOWLEDGEMENT

The authors would like to acknowledge the support of the Cancer Research Campaign, UK.

### REFERENCES

Chan HSL, Thorner PS, Haddad G and Ling V (1990) Immunohistochemical detection of P-glycoprotein: prognostic correlation in soft tissue sarcoma of childhood. *J Clin Oncol* **8:** 689–704

Cowie F, Pinkerton CR, Philips M, Dick G, Judson I, McCarthy PT and Flanagan, RJ (1995) Continuous-infusion verapamil with etoposide in relapsed or resistant paediatric cancer. *Br J Cancer* **71:** 877–881

Dalton WS, Grogan TM, Meltzer PS, Scheper RJ, Durie BGM, Taylor CW, Miller TP and Salmon SE (1989) Drug-resistance in multiple myeloma and non-Hodkin's lymphoma: detection of P-glycoprotein and potential circumvention by addition of verapamil to chemotherapy. *J Clin Oncol* **7:** 415–424

Durie BGM and Dalton WS (1988) Reversal of drug-resistance in multiple myeloma with verapamil. *Br J Haematol* **68:** 203–206

Eisenhauer EA, ten Bokkel Huinink WW, Swenerton KD, Gianni L, Myles J, van der Burg MEL, Kerr I, Vermorken JB, Buser K, Colombo N, Bacon M, Santabarbara P, Onetto N, Winograd B and Canetta R (1994) European–Canadian randomized trial of paclitaxel in relapsed ovarian cancer: high-dose versus low-dose and long versus short infusion. *J Clin Oncol* **12:** 2654–2666

Gianni L, Kearns CM, Giani A, Capri G, Vigano L, Locatelli A, Bonadonna G and Egorin MJ (1995) Nonlinear pharmacokinetics and metabolism of paclitaxel and its pharmacokinetic/pharmacodynamic relationships in humans. *J Clin Oncol* **13:** 180–190

Hodgson ST, (1994) Discovery of 1069C- a novel synthetic antitumour agent with low cross-resistance potential. In *Medicinal Chemistry: Principles and Practice*, King FD (ed.), p. 241. The Royal Society of Chemistry: Cambridge

Hodgson ST, Jenkins DC, Knick V, Rapson EB and Watts SDM (1992) Synthetic and biological properties of 1069C: a new synthetic antitumour agent with very low cross resistance potential. *Bioorg Med Chem Lett* **34:** 1257–1264

Miller TP, Grogan TM, Dalton WS, Spier CM, Scheper RJ and Salmon, SE (1991) P-glycoprotein expression in malignant lymphoma and reversal of clinical drug resistance with chemotherapy plus high-dose verapamil. *J Clin Oncol* **9:** 17–24

Raynaud F, Walton M and Judson I (1993) An HPLC assay for the measurement of 1069C85 in biological tissue and fluids. *J Chromatogr Biomed Appl* **622:** 243–248

Raynaud FI, Kelland LR, Walton MI and Judson IR (1994) Preclinical pharmacology of 1069C85, a novel tubulin inhibitor. *Cancer Chemother Pharmacol* **35:** 169–173

Rowinsky EK, Noe DA, Grochow LB, Bowling MK, Sartorious SE, O'Reilly S, Chen T and Donehower RC (1995) Phase I and pharmacological study of CI-980, a synthetic and structurally unique antimicrotubule agent, on a 72-hour continuous infusion schedule in adults with solid tumors. *Proc Am Soc Clin Oncol* **14:** 459

Sklarin NT, Benson L, Roca J, Einzig AI, Wiernik PH, Whitfield LR, Lathia C, Kowal C and Grove W (1995) Phase I study of CI-980: 24-hr infusion schedule. *Proc Am Soc Clin Oncol* **14:** 479

Sonneveld P, Durie BGM, Lokhorst HM, Marie JP, Solbu G, Suciu S, Zittoun R, Lowenberg B and Nooter K (1992) Modulation of multidrug-resistant multiple myeloma by cyclosporin. *Lancet* **340:** 255–259

van Asperen J, Schinkel AH, Beijnen JH, Nooijen WJ, Borst P and van Tellingen O (1996) Altered pharmacokinetics of vinblastine in mdrla P-glycoprotein-deficient mice. *J Natl Cancer Inst* **88:** 994–999

© Cancer Research Campaign 1997